# Osteosynthesis using a scorpion plate and a shaped bone graft for distal clavicle nonunion with bone defect: a case report

**DOI:** 10.1093/jscr/rjaf869

**Published:** 2025-11-05

**Authors:** Takeaki Hashimoto, Ryogo Furuhata, Taiki Sato, Yusuke Shiba, Noboru Matsumura, Atsushi Tanji

**Affiliations:** Department of Orthopaedic Surgery, Ashikaga Red Cross Hospital, 284-1 Yobe-cho, Ashikaga-shi, Tochigi 326-0843, Japan; Department of Orthopaedic Surgery, Ashikaga Red Cross Hospital, 284-1 Yobe-cho, Ashikaga-shi, Tochigi 326-0843, Japan; Department of Orthopaedic Surgery, Ashikaga Red Cross Hospital, 284-1 Yobe-cho, Ashikaga-shi, Tochigi 326-0843, Japan; Department of Orthopaedic Surgery, Ashikaga Red Cross Hospital, 284-1 Yobe-cho, Ashikaga-shi, Tochigi 326-0843, Japan; Department of Orthopaedic Surgery, Keio University School of Medicine, 35 Shinanomachi, Shinjuku-ku, Tokyo, 160-8582, Japan; Department of Orthopaedic Surgery, Ashikaga Red Cross Hospital, 284-1 Yobe-cho, Ashikaga-shi, Tochigi 326-0843, Japan

**Keywords:** nonunion, distal clavicle fracture, bone defect, bone graft, scorpion plate

## Abstract

Unstable distal clavicle fractures often develop symptomatic nonunion following conservative treatment; however, cases in which bone defect occurs at the site of nonunion are rare. We report a case of distal clavicle nonunion with a bone defect that was treated with osteosynthesis using a scorpion plate and a shaped bone graft. A 66-year-old man presented with symptomatic distal clavicle nonunion sustained 5 months earlier. We reduced the fracture fragments, revealing a bone defect at the nonunion site. We harvested a bicortical iliac bone graft with a triangular column shape to match the shape of the bone defect and used a scorpion plate for fixation. Postoperatively, bone union and the satisfactory functional outcomes were achieved. Thus, our technique is advantageous in cases of distal clavicle nonunion with bone defect. Using the scorpion plate enabled fixation of the bone graft without further fragmentation, while shape-matched bone grafting to the defect restored clavicle length.

## Introduction

Nonunion of the distal clavicle is a complication of unstable, displaced distal clavicle fractures, and occurs with a frequency of 22%–50% [1, 2]. The distal clavicle nonunion often presents with pain, muscle weakness, and limited range of motion, necessitating surgery [3]. Plate fixation with cancellous bone grafting yields high rates of bone union and satisfactory functional outcomes for symptomatic nonunion of the distal clavicle [4–8]. However, cases in which the bone defect occurs at the site of nonunion are extremely rare [4].

The scorpion plate (Aimedic MMT Co., Ltd., Tokyo, Japan) is an anatomical nonlocking plate. In addition to two screws, the plate arms can hold the distal bone fragment. Osteosynthesis using the scorpion plate has achieved high bone union rate [9–11] and satisfactory shoulder functional outcomes [11] in Neer Type II or V distal clavicle fractures. Furthermore, a recent case report [8] described the satisfactory outcomes achieved using scorpion plate fixation and cancellous bone grafting for distal clavicle nonunion with small distal bone fragments.

Herein, we report a case of distal clavicle nonunion with a bone defect that was treated with plate fixation using a scorpion plate and an iliac bone graft shaped like a triangular column.

## Case report

**Figure 1:**
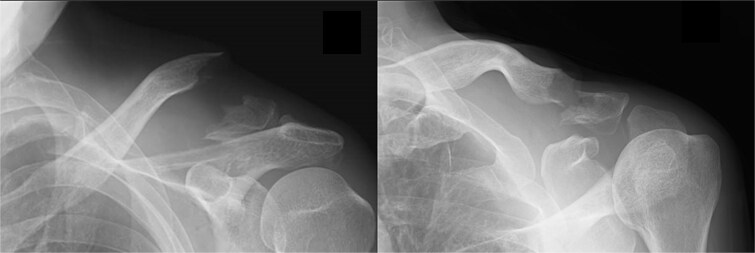
Radiographs captured immediately after injury show a fracture of the left distal clavicle with displacement.

**Figure 2:**
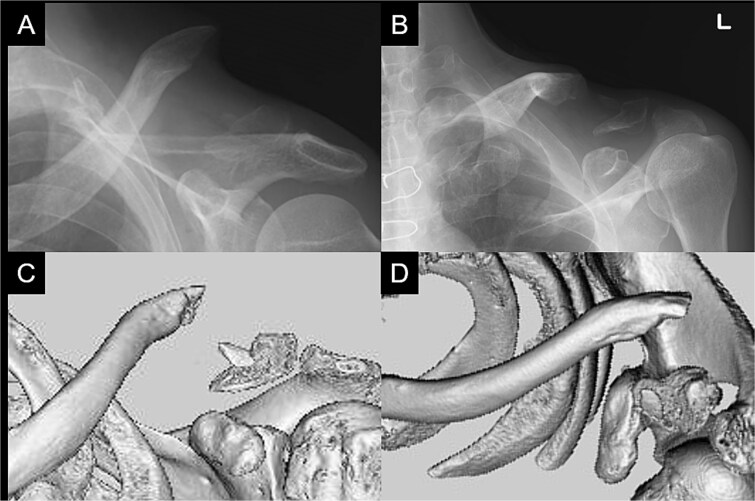
Radiographs (A and B) and CT scans (C and D) captured after 5 months of conservative treatment show that the displacement has progressed.

A 66-year-old man presented with left shoulder pain after falling. A Neer Type IIB fracture of the distal clavicle with significant displacement was revealed on radiographs (Fig. 1); however, owing to severe aortic valve stenosis and heart failure, the patient was deemed unfit for surgery. The patient underwent conservative treatment for distal clavicle fracture for 5 months; however, the pain in the left shoulder persisted, and significant skin protrusion caused by a displaced bone fragment was noted. No findings suggestive of neurovascular injury were observed. Radiography and computed tomography (CT) examinations revealed fracture displacement progression (Fig. 2A–C). After undergoing aortic valve replacement 3 months postinjury, the cardiac function of the patient improved and surgery was performed to treat the distal clavicle nonunion.

**Figure 3:**
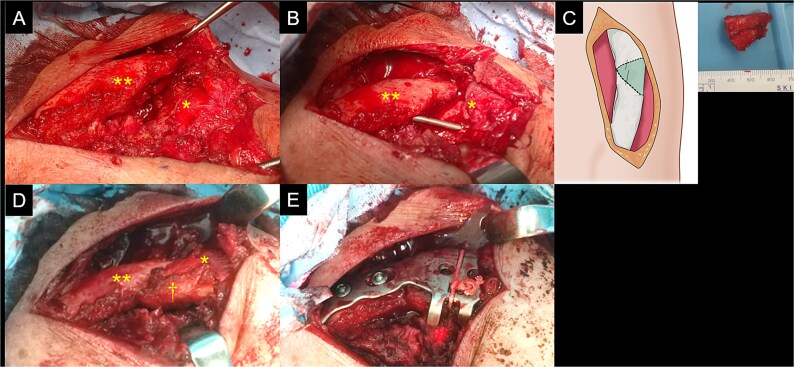
Intraoperative findings of the left clavicle. The proximal (double asterisk) and distal (asterisk) fragments (A) at the site of nonunion are exposed. After being reduced, the fragments are fixed using a Kirschner wire and a triangular column-shaped bone defect is identified at the site of nonunion (B). A shape-matched bicortical bone graft is harvested from the iliac wing (C). After bone grafting (dagger) (D), a scorpion plate is used for fixation (E).

We performed the surgery with the patient in the beach chair position under general anaesthesia. We made a longitudinal 8-cm incision in the skin on the distal clavicle. We removed the fibrous tissue at the nonunion site (Fig. 3A), and the medullary canal of the fracture site was opened using a Kirschner wire. After reducing the proximal and distal fragments and temporally fixing them using a Kirschner wire, we identified a triangular column-shaped bone defect (2 cm) at the nonunion site (Fig. 3B). We harvested a bicortical bone graft with a triangular column shape from the iliac wing to match the shape of the bone defect (Fig. 3C). After bone grafting (Fig. 3D), we performed plate fixation using a scorpion plate (Fig. 3E).

**Figure 4:**
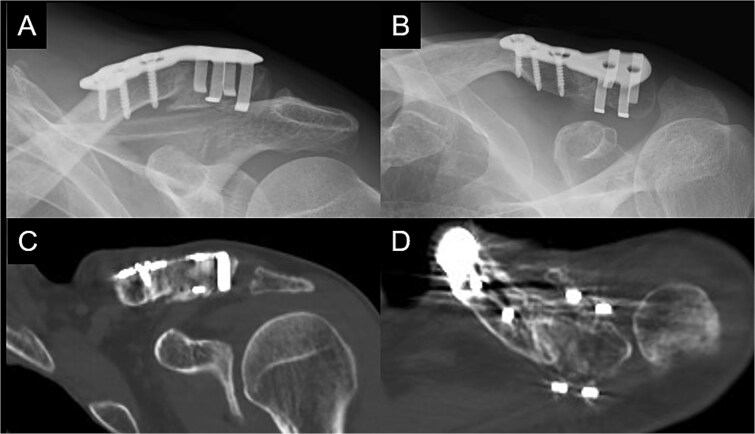
Radiographs (A and B) and CT scans (C and D) captured at 1 year postoperatively show union of the bone.

Postoperatively, the left arm of the patient was immobilized in a sling for 3 weeks. The pain gradually subsided, and the patient resumed heavy work without pain at 6 months postoperatively. At 1 year postoperatively, the patient had a constant score of 98. Bone union was confirmed (Fig. 4A–D).

## Discussion

In the present case, osteosynthesis using a scorpion plate and triangular column-shaped iliac bone graft was used to treat a patient with nonunion of the distal clavicle with a bone defect. The surgery resulted in a solid bone union and satisfactory postoperative functional outcomes. This case highlights two clinical issues.

First, in distal clavicle nonunion with bone defects, using bicortical or tricortical bone grafts shaped to match the bone defect can be advantageous for achieving bone union and restoring the clavicle length. The use of bone grafting in surgery for nonunion of the clavicle remains controversial [12, 13]. However, restoring the length of the clavicle is associated with an improved constant score [5], while using a tricortical bone graft corrects the clavicle length in cases of nonunion [14]. Therefore, tricortical bone grafting is generally indicated for atrophic clavicle nonunion with a bone defect or shortening deformity [5]. When nonunion occurs in the midshaft of the clavicle, a shortening deformity often develops; tricortical bone grafting tailored to the shape of the nonunion site has achieved solid bone union and clavicle length restoration [15, 16]. However, nonunion of the distal clavicle with bone defects at the site of nonunion are extremely rare. In most cases, high rates of bone union are achieved with cancellous bone grafting [4–8]. In the only reported case of distal clavicle nonunion with a large (1.5 cm) bone defect, plate fixation with tricortical iliac bone grafting was performed, resulting in bone union and clavicle length restoration [4]. In the present case, a triangular bone defect was identified at the site of the nonunion, and a shape-matched bicortical iliac bone graft was used to achieve anatomical reduction and bone union.

Second, in distal clavicle nonunion with small distal fragments and bone defect, the scorpion plate enables the secure fixation of the distal fragment and bone graft while avoiding further fragmentation. To date, plate fixation and cancellous bone grafting have shown satisfactory postoperative outcomes in distal clavicle nonunion [4–8]; however, because the fixation strength of the distal fragment depends on the number of distal screws used, small distal fragments of < 2 cm in length are associated with poor outcomes [4]. In the only reported case in which plate fixation was combined with tricortical bone grafting [4], the distal and graft bone fragments were fixed using multiple locking screws, soft wires, and a positioning screw, achieving bone union. With the scorpion plate, the distal bone fragment and bone graft can be fixed using two plate arms instead of multiple screws [8]; thus, further fragmentation is avoided, despite the surgical technique being relatively simple. This is a major advantage of using the scorpion plate. Therefore, the scorpion plate may be a suitable treatment option for distal clavicle nonunion with small distal bone fragments and bone defects.

In summary, this report provides new surgical insights on distal clavicle nonunion with bone defect. The scorpion plate enables the secure fixation of the distal bone fragment and bone graft without further fragmentation, while shape-matched bone grafting can restore the clavicle length.

## Data Availability

Data supporting the findings of this study are available from the corresponding author upon request.

## References

[1] Nordqvist A, Petersson C, Redlund-Johnell I. The natural course of lateral clavicle fracture. 15 (11-21) year follow-up of 110 cases. Acta Orthop Scand 1993;64:87–91. 10.3109/17453679308994539 8451958

[2] Robinson CM, Cairns DA. Primary nonoperative treatment of displaced lateral fractures of the clavicle. J Bone Joint Surg Am 2004;86:778–82. 10.2106/00004623-200404000-00016 15069143

[3] Jupiter JB, Leffert RD. Non-union of the clavicle. Associated complications and surgical management. J Bone Joint Surg Am 1987;69:753–60. 10.2106/00004623-198769050-00018 3597476

[4] Kang HJ, Kim HS, Kim SJ, et al. Osteosynthesis of symptomatic nonunions of type II fractures of the distal clavicle using modified locking T-plate and bone grafting. J Trauma Acute Care Surg 2012;72:E14–9. 10.1097/TA.0b013e31822fb987 22327996

[5] Schnetzke M, Morbitzer C, Aytac S, et al. Additional bone graft accelerates healing of clavicle non-unions and improves long-term results after 8.9 years: a retrospective study. J Orthop Surg Res 2015;10:2. 10.1186/s13018-014-0143-y 25573541 PMC4296679

[6] Santolini E, Stella M, Sanguineti F, et al. Treatment of distal clavicle nonunion with and without bone grafting. Injury 2018;49:S34–8. 10.1016/j.injury.2018.11.016 30518508

[7] Tenpenny W, Caldwell PE 3rd, Rivera-Rosado E, et al. Arthroscopic-assisted bone graft harvest from the proximal humerus for distal third clavicle fracture nonunion. Arthrosc Tech 2020;9:e1937–42. 10.1016/j.eats.2020.08.018 33381403 PMC7768109

[8] Yokoyama Y, Furuhata R, Tanji A, et al. Osteosynthesis using scorpion plate for nonunion of distal clavicle fracture with small distal bone fragment: a case report. Trauma Case Rep 2023;48:100953. 10.1016/j.tcr.2023.100953 37876980 PMC10590729

[9] Furuhata R, Takahashi M, Hayashi T, et al. Treatment of distal clavicle fractures using a scorpion plate and influence of timing on surgical outcomes: a retrospective cohort study of 105 cases. BMC Musculoskelet Disord 2020;21:146. 10.1186/s12891-020-3169-9 32131803 PMC7057610

[10] Furuhata R, Takahashi M, Matsumura N, et al. Osteosynthesis using the anatomical plate with grasping arms for unstable distal clavicle fractures: a technical trick and clinical experience. J Orthop Trauma 2021;35:e263–7. 10.1097/BOT.0000000000001922 33771960

[11] Furuhata R, Matsumura N, Kamata Y, et al. Osteosynthesis using scorpion plate for Neer type V distal clavicle fracture. BMC Musculoskelet Disord 2024;25:909. 10.1186/s12891-024-08039-z 39543629 PMC11566308

[12] Ramoutar DN, Rodrigues J, Quah C, et al. Judet decortication and compression plate fixation of long bone non-union: is bone graft necessary? Injury 2011;42:1430–4. 10.1016/j.injury.2011.03.045 21497808

[13] Rollo G, Vicenti G, Rotini R, et al. Clavicle aseptic nonunion: is there a place for cortical allogenic strut graft? Injury 2017;48:S60–5. 10.1016/S0020-1383(17)30660-5 29025612

[14] Kirchhoff C, Banke IJ, Beirer M, et al. Operative management of clavicular non-union: iliac crest bone graft and anatomic locking compression plate. Oper Orthop Traumatol 2013;25:483–98. 10.1007/s00064-013-0257-0 24061703

[15] Simpson NS, Jupiter JB. Clavicular nonunion and malunion: evaluation and surgical management. J Am Acad Orthop Surg 1996;4:1–8. 10.5435/00124635-199601000-00001 10790675

[16] Furuhata R, Yokoyama Y, Tanji A, et al. Plate fixation using parallelogram prism iliac bone grafts for clavicle oblique nonunion with shortening deformity: a case report. BMC Musculoskelet Disord 2023;24:346. 10.1186/s12891-023-06468-w 37143033 PMC10157972

